# An Ensemble of Long Short‐Term Memory Models to Automatically Detect End‐Range Movement Patterns in Men's Professional Hard Court Grand Slam Tennis

**DOI:** 10.1002/ejsc.70081

**Published:** 2026-03-26

**Authors:** Cameron Armstrong, Peter Peeling, Alistair Murphy, Simon Denman, Machar Reid

**Affiliations:** ^1^ School of Human Sciences (Exercise and Sport Science) The University of Western Australia Perth Australia; ^2^ Tennis Australia Melbourne Australia; ^3^ Western Australian Institute of Sport Perth Australia; ^4^ School of Electrical Engineering and Robotics Queensland University of Technology Brisbane Australia

**Keywords:** load management, machine learning, pose data, skeletal tracking

## Abstract

Evaluating end‐range movements during tennis match‐play can quantify high intensity load exposure and facilitate specific analysis of players' high‐end physical capabilities. Currently, the process to evaluate such movement is labour intensive, with an established and efficient process to identify end‐range movements lacking. Using three‐dimensional pose model data for male competitors in the 2024 Australian Open, we evaluated an ensemble of long short‐term memory (LSTM) models to correctly classify coach‐identified end‐range movement patterns. An ensemble of 10 LSTM models that took the average prediction value and applied a class prediction threshold of 0.63 was the best performing approach, producing an F1‐score of 0.944, overall accuracy of 95.9%, precision of 97.8% and recall of 91.2%. From these results, we provide a novel and practical way of using real‐world pose model data and machine learning to automatically detect one of the most physically demanding movement tasks in professional men's tennis. This work enhances post‐match analysis via an automated analytical pipeline that can quantify high intensity movement exposures and produce descriptive statistics of end‐range movement to assist with the load monitoring and management of professional players.

## Highlights

- An ensemble of LSTM models can detect end‐range tennis movements with high accuracy, precision and recall
- An ensemble of LSTM models produces higher F1‐scores than similar random forest classification tasks on tennis movements
- This automated detection of end‐range movements has implications for load monitoring in tennis

## 1 Introduction

Professional tennis demands a mix of speed, acceleration, deceleration, and change of direction (CoD) qualities from players (Armstrong et al. 2024a; Carvalho et al. 2014; Giles et al. 2019). For decades, quantifying these demands during competition was a challenge, yet the advance of computer science models has driven the collection of tracking data in the field that can be examined to understand player movement in increasingly novel ways (Armstrong et al. 2024a; M. Kovacs et al. 2007; Pluim et al. 2023; Armstrong et al. 2024b). For example, K‐means clustering was recently employed to identify six general movement patterns based on the tracking data of male players during Grand Slam competition on hard courts (Armstrong et al. 2024a). Although match summaries describing total distance covered (among other variables) are still useful, analysing movement at the shot level is appealing to account for an athlete's performance under specific conditions. Indeed, Armstrong, Peeling, Murphy and Reid (Armstrong et al. 2024a) describe the distance, direction, speed, time pressure and acceleration profile of each movement pattern in their findings, providing substantial detail and context for professional tennis movement requirements. Further, Lateral End‐Range Red (LERR) was considered the most physically demanding movement pattern during match play (Armstrong et al. 2024a) where better ranked athletes were observed to outperform lesser ranked players during LERR specifically (Armstrong et al. 2025). The LERR movement pattern demonstrates that superior physical ability allows for better shot outcomes and highlights the importance of physical ability in tennis competition (Armstrong et al. 2025).

Given its importance, detailed analysis of LERR from competition could lead to targeted training interventions for performance enhancement (Giles et al. 2019). However, quantifying and evaluating end‐range movements remains labour‐intensive, often necessitating manual notation (Giles et al. 2019), making the quantification of these important movements during competition hard to obtain. Of note, the K‐means algorithm employed in previous classification literature is limited when identifying individual movements such as LERR but are useful in uncovering common movement patterns from a larger dataset (Al‐jabery et al. 2019a). Indeed, a known limitation of the K‐means approach is its broad and spherical classification patterns which complicates the precise categorisation of tennis movement cycles more specifically (Al‐jabery et al. 2019a). As such, other machine learning approaches could be better suited to identifying singular movements such as LERR (Al‐jabery et al. 2019a, 2019b; Ward et al. 2021), yet their utility is constrained when interpreting only centre of mass information in two dimensions.

Previously, markerless motion capture measuring two‐dimensional positions of the centre of mass has been used to quantify competition demands in tennis (Armstrong et al. 2024a; Pluim et al. 2023). More recently, pose model data have been made available which details three‐dimensional joint locations for athletes during competition. These joint locations include shoulders, elbows, wrists, hips, knees and ankles (amongst others). Several biomechanical statistics derived from this competition pose data have been used for broadcast purposes (Trollope 2024), but more generally, these data proffer the opportunity to quantify key movement attributes, such as balance and stride characteristics (i.e., stride length and stride frequency) (Carvalho et al. 2014), direct from competitive performance. Such a rich data source could also provide additional information of movement kinematics from competition, ultimately useful in categorising movement cycle instances into the described movement patterns of recent research with a suitable machine learning approach (Al‐jabery et al. 2019a, 2019b; Ward et al. 2021).

The enrichment of joint position data may provide enough kinematic detail from an athlete's movement during competition to improve single instance categorisation of movement cycles during match performances, although a different machine learning algorithm more suited to this specific classification task warrants consideration (Al‐jabery et al. 2019a, 2019b; Ward et al. 2021; Giles et al. 2020, 2024; Giles et al. 2023; Wei et al. 2016). Recently, a random forest model was trained to identify CoD instances within tennis competition using centre of mass data (F1‐score of ∼73%) (Giles et al. 2020), before being used to profile CoD demands of elite tennis competition (Giles et al. 2024). Further, this random forest model was used to classify CoD instances, where the results were combined with hierarchical clustering to identify player ‘styles’ during CoD tasks in tennis competition (Giles et al. 2023). Although random forest models have been shown to effectively classify discrete movement instances in tennis competition, long short‐term memory (LSTM) models may be more effective owing to their ability to ‘remember’, ‘forget’ and ‘output’ relevant information when considering sequential data (Al‐jabery et al. 2019b). Although deep learning models (such as LSTM models) tend to overfit and exhibit over‐confidence (problematic for real‐world applications), an ensemble approach (i.e., training multiple LSTM models which ultimately contribute to a predicted class) addresses such tendencies (Al‐jabery et al. 2019b; Ward et al. 2021). Ensembles also present superior classification accuracy when compared to single LSTM models when using motion‐based time series data (Ward et al. 2021).

In summary, prior research shows that the LERR movement cycle in professional tennis can distinguish better ranked players and outlines the most challenging physical demands of competition. However, identifying LERR movement cycles from competition data is a real‐world challenge, where solving such a problem would allow large scale movement evaluation for practitioners working with professional tennis athletes. As such, the aim of this investigation was to describe the most effective ensemble of LSTM models to automatically classify LERR movement cycles of tennis athletes using novel pose model tracking data from professional tennis competition.

## 2 Materials and Methods

### 2.1 Participants

All main draw Australian Open male participants who competed on Rod Laver Arena during the 2024 Australian Open were considered for this study (*n* = 27). Participant consent was obtained on entry into the Australian Open tournament and an institutional research ethics committee provided ethical approval for the study (2022/ET000216).

### 2.2 Data Collection

Participants competed during the 2024 Australian Open Grand Slam tournament in live match‐play, where key joint positions (X, Y, and Z coordinates) and ball positions (X, Y, and Z coordinates) were recorded using Hawk‐Eye technology (Hawk‐Eye Innovations Ltd, Basingstoke, UK). Hawk‐Eye uses a system of 10 cameras to track ball coordinates in tennis match‐play with a mean error of 2.6 mm (Innovations 2016) and provides processed and filtered three‐dimensional coordinate data. Within these data, individual movement cycles were identified (as detailed in previous work (Armstrong et al. 2024a, 2024b, 2025)) with the moment of opposition hitting impact used as a surrogate for the split‐step (Mecheri et al. 2019). At the end of a rally, the final movement cycle was terminated when one of the following conditions was met:

1. The ball impacted the net and did not land within the field of play (net error)
2. 0.5 s after any other error (general error)
3. 0.5 s after the bounce of a successful final shot (winner)

Data were then checked for errors associated with tracking drop out (i.e., a player leaving the Hawk‐Eye camera calibration volume). Where an error was identified in a movement cycle, the entire rally data file and all associated movement cycle information was removed for both participants. This resulted in 4284 movement cycles available for analysis.

The associated broadcast footage for each movement cycle was identified and saved as an individual video clip. These individual movement cycle clips were then randomly allocated into playlists of 20 clips for evaluation. Tennis movement experts (*n* = 3) with > 10 years' experience in tennis on average, were then tasked with identifying the LERR movement cycles within each playlist, based on the definition available in literature (Armstrong et al. 2024a). An initial pilot of rater agreement was conducted using the LERR movement cycle definition, where tennis movement experts were asked to rate any movement cycle that met the LERR definition as ‘yes’ and ‘no’ if not. Initially, unacceptable agreement was achieved, and examples where disagreement occurred were explored further. After three iterations of this process an additional ‘partial’ rating was included to allow for ambiguity, and a further three criteria were added to the end‐range requirements:

1. High intensity movement (defined as high stride frequency or long stride length) during the entire movement cycle
2. A CoD effort
3. A cross‐over step to initiate recovery once the player looked able to recover from reaching the ball

This classification criteria allows for raters to class clear end‐range movement cycles with confidence, allows a category for those end‐range examples that are somewhat unclear, and provides a third classification group for those examples that are most definitely not end‐range. Finally, a subset of 5 playlists (100 individual movement cycle clips) were used to evaluate intra‐ and inter‐rater agreement within and between expert raters, respectively. The minimum required number of observations for appropriate rater agreement statistical power was 76 (Rotonda 2022). Intra‐rater agreement intraclass correlation coefficient (ICC) values ranged between 0.98–1.00 and the inter‐rater agreement kappa coefficient value was 0.93. The playlists of 20 movement cycle clips were then divided between the raters and each movement cycle was evaluated. This resulted in 407 movement cycles identified as end‐range (‘yes’), 576 movement cycles with end‐range characteristics but not all criteria met (‘partial’), and 3301 movement cycles that were not end‐range (‘no’). To provide further clarity to the ensemble and differentiate classes more clearly, the ‘partial’ examples were removed from training. The classification ensemble is, therefore, trained on the best examples of end‐range movement cycles according to the expert raters. Ultimately, this means the partial class will be determined as end‐range or not end‐range based on the dominance of end‐range features within each instance.

### 2.3 Data Transformation

Once movement cycle ratings were collected, the associated Hawk‐Eye joint positional data were extracted accordingly. The joint positions of the shoulders (left and right X, Y, and Z) and hips (left, mid, and right X, Y, and Z) were included, as it is expected these joint locations are less variable than more distal limb joint coordinates during tennis movement tasks (Giles and Reid 2021). Furthermore, the features were extracted pertaining to distance, velocity, and acceleration in one‐dimension (i.e., X, Y, *or* Z), two‐dimensions (i.e., X *and* Y), and three‐dimensions (i.e., X, Y, *and* Z), and were added to the dataset for each movement cycle. Distance was defined as the difference between axis positions (in metres) for each observation in a sample by calculating a one‐, two‐, or three‐dimensional scalar. Velocity was calculated by dividing each distance value (one‐, two‐, or three‐dimensional distance) by the sampling time between each observation (0.02 s in the case of Hawk‐Eye sampling at 50 Hz). Acceleration was calculated by dividing the change in each velocity (one‐, two‐, or three‐dimensional velocity) by the sampling time between observations in each sample. This meant that for each of the five joint positions (i.e., left shoulder, right shoulder, left hip, mid hip, and right hip), an additional one‐, two‐, and three‐dimensional distance, velocity, and acceleration feature was available. The data associated with each movement cycle was then time‐normalised by transforming the sequence of observations to a time scale of 0–1 (i.e., 0%–100% completion), where 0 was the start of the movement cycle and 1 was the end of the movement cycle. The data sequence was incremented by an interval of 0.01 and missing data were imputed using linear 1‐dimensional interpolation for each available feature (using the ‘interp1d’ function from the sciPy module [version 1.12.0]). This resulted in a data file for each observation with a uniform sequence length of 100 samples and all original data preserved.

To account for class imbalance, all ‘yes’ observations were oversampled four‐fold, as oversampling is considered best practice for deep learning class‐imbalanced classification tasks (Buda et al. 2018). The data were initially doubled by down sampling each observation to a length of 50 by taking every odd index sample and every even index sample, resulting in an odd sample observation and an even sample observation. To double the data yet again and achieve four‐fold oversampling, each sample had the *x*‐axis and *y*‐axis inverted, which does not distort the movement profile in anyway but rather reflects the same movement from the opposite end of the court (i.e., a forehand on one end of the court is inverted to reflect the same forehand at the other end of the court). Each sample in the four‐fold oversampling method is described below:

1. Every *odd* index point (i.e., point 1, point 3, etc.) with *original x*‐axis and *y*‐axis values
2. Every *odd* index point (i.e., point 1, point 3, etc.) with *inverted x*‐axis and *y*‐axis values
3. Every *even* index point (i.e., point 2, point 4, etc.) with *original x*‐axis and *y*‐axis values
4. Every *even* index point (i.e., point 2, point 4, etc.) with *inverted x*‐axis and *y*‐axis values

This resulted in 1628 end‐range samples (33%) and 3301 non‐end‐range samples (67%) in the final dataset. Data were split into training (70%) and testing (30%) sets using the ‘train_test_split’ function from the sci‐kit learn module (version 1.4.1.post1). Because the values of the X, Y, and Z coordinates and engineered features were of different magnitudes or scales, all individual features were transformed to z‐scores to normalise the data. The ‘StandardScaler’ function from the sci‐kit learn module (version 1.4.1.post1) was used for data normalisation. The scaler was fit on the training dataset to prevent information leakage and was then used to transform both the training and testing data sets. To further handle the persisting class imbalance, class weights were computed using the ‘compute_sample_weight’ function from the sci‐kit learn module (version 1.4.1.post1), which were calculated using the training dataset. The weighting for a ‘yes’ class was 1.496 and for a ‘no’ class was 0.751.

### 2.4 Statistical Analysis

An ensemble of long short‐term memory (LSTM) recurrent neural network (RNN) models were trained and utilised to evaluate the precision and recall by the F1‐score in this investigation. Precision is a metric that determines the ability of a model to correctly identify positive instances (i.e., correctly identify end‐range in this case), where higher rates of false positive predictions are penalised. Recall is a metric detailing the model's ability to correctly identify all actual positive instances, where higher rates of false negatives are penalised. The F1‐score is the harmonic mean between the precision and recall which is used to understand the overall reliability of a model. TensorFlow (version 2.16.1) and keras (version 3.3.3) modules were used to build, train and evaluate these models. The ensemble contained 10 models, each consisting of four sequential layers:

1. A bi‐directional LSTM (Bi‐LSTM) layer with 128 units which returned sequences
2. A LSTM layer with 128 units which returned only the final embedding
3. A dense layer with 64 units and a rectified linear unit (ReLU) activation function
4. A dense layer with 1 unit and a sigmoid activation function

Each model in the ensemble was compiled with the binary cross‐entropy loss function and the Adam optimiser (with a learning rate of 0.001). The validation split was set to 20% and the batch‐size was set at 32. The evaluation metric for training validation was binary cross‐entropy. Early stopping was specified by using the validation loss, with patience set to three and the mode set as the minimum, with models trained for a maximum of 20 epochs. Once the 10 models were trained on the training data, each model was evaluated on the testing data. Several aggregation procedures were explored with different conditions to determine the predicted class. For every sample in the test dataset, each of the 10 models' individual predictions (i.e., model 1 prediction for test sample 1, model 2 prediction for test sample 1, etc.) were compared against a threshold between 0.5 and 0.9 in 0.01 increments (i.e., 0.50, 0.51, etc.). If the condition was met, the prediction class was positive (‘yes’), otherwise it was classed negative (‘no’). Once all test samples were evaluated, the number of positive class predictions was summed for each test sample (i.e. 6 models predict ‘yes’ for sample 1), where a minimum positive class count was further evaluated, providing a result for the overall ensemble class prediction. A minimum positive class count between 6 and 10 were evaluated, which resulted in 205 evaluations (41 threshold combinations and 5 model count combinations). In addition to this aggregation approach, the mean prediction value of all 10 models for each sample was compared against a threshold between 0.5 and 0.9 in 0.01 increments. If the mean prediction value for all 10 models met or exceeded the threshold, the ensemble prediction for that test sample was positive (‘yes’), otherwise it was classed negative (‘no’). This resulted in 41 evaluations (41 threshold combinations and 1 mean model prediction value) in this aggregation procedure. The F1‐scores were then computed for each aggregation procedure, where the highest F1‐score was identified to determine the highest performing ensemble aggregation method.

## 3 Results

**FIGURE 1:**
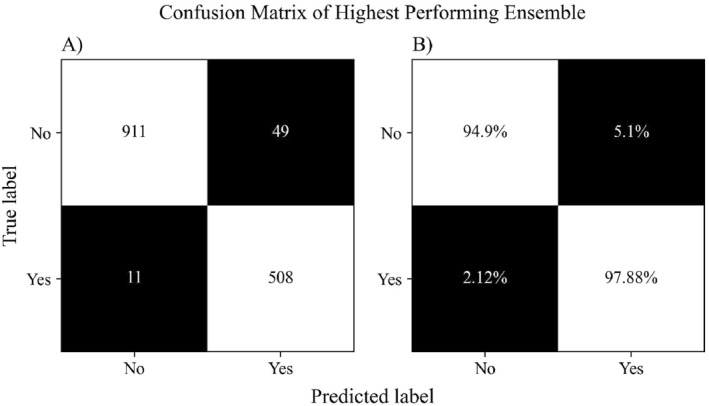
Absolute (A) and relative (B) confusion matrix of the average score ensemble with a classification threshold of 0.63.

**FIGURE 2:**
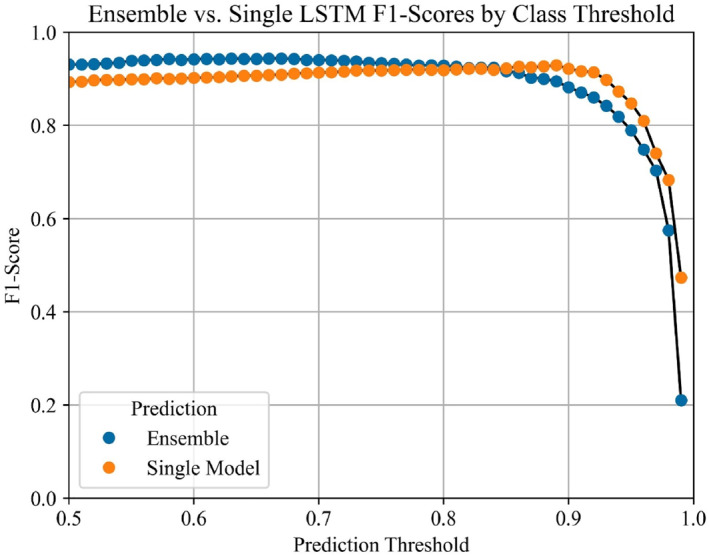
Comparison of F1‐scores across each investigated threshold between a single LSTM and an ensemble of 10 LSTM models.

**FIGURE 3:**
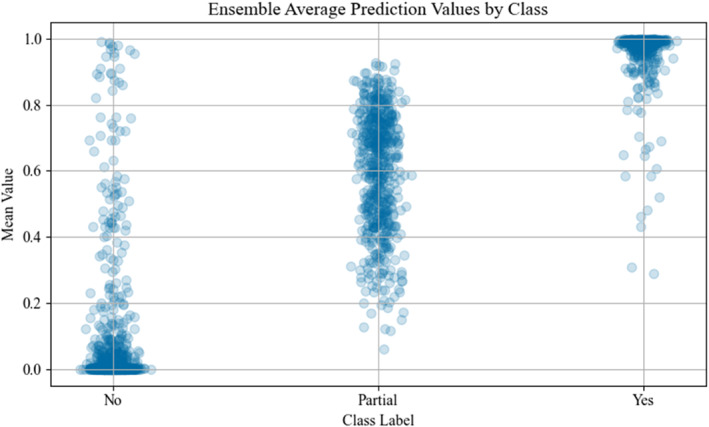
Ensemble average prediction scores from testing stage, including partial class data.

**FIGURE 4:**
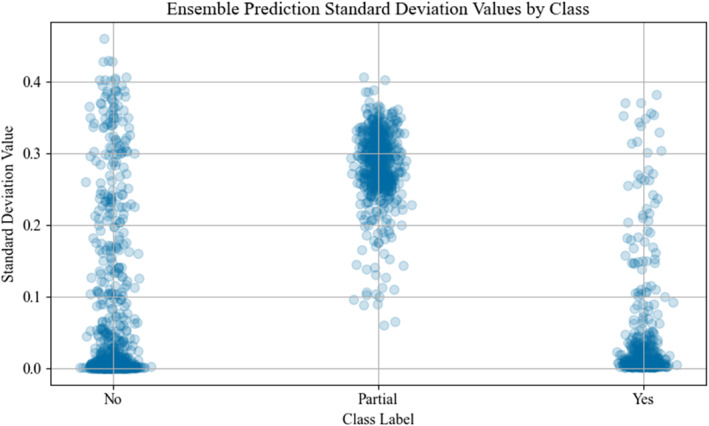
Standard deviation values for each LSTM model within the ensemble from the testing stage, including partial class data.

Each model in the ensemble contained 429,697 trainable parameters. The highest F1‐score was 0.944 which was produced by the second aggregation procedure (i.e., average prediction of all model values) with a threshold of 0.63. This approach also resulted in an overall accuracy of 95.9%, precision of 97.8%, and recall of 91.2%. Figure 1 shows the confusion matrix for this approach, both in absolute (Figure 1A) and relative (Figure 1B) values. An average inference time of 1.01 s was obtained to process a movement cycle from a match when running on an Intel Core i71165G7 system with no GPU acceleration. This allowed the complete match of 318 cycles to be processed in 5 min and 21 s. Figure 2 shows the comparison of F1‐scores for the best aggregation procedure against a single LSTM model at each threshold interval. Analysis of the testing dataset also included the annotated labels of ‘partial’ to evaluate the model further, where Figure 3 shows the overall ensemble prediction score (i.e., average of all 10 LSTM model values) from the expanded testing dataset, and Figure 4 shows the standard deviation of the 10 model prediction scores.

## 4 Discussion

This investigation examines the use of LSTM models with novel pose model data to automatically classify LERR movement cycles from male Grand Slam tennis competition on hard court surfaces. The best performing approach consisted of 10 LSTM models (ensemble) using the average value of each LSTM model with a threshold of 0.63. The approach produced an F1‐score of 0.944, supporting the use of this approach for accurately identifying individual LERR movement cycle instances from competition. The accuracy of 95.9% suggests our approach is successful in identifying almost all LERR instances, supported by a precision of 97.8% (indicating a low rate of false positives) and a recall of 91.2% (indicating a low rate of false negatives). These findings provide practitioners with confidence that occasions of LERR movement can be accurately identified with minimal misclassification.

The use of LSTM models is novel as a classification method in sport, particularly when classifying complex multi‐dimensional movements such as tennis movement cycles. Overall, the range of ensemble average values (present in Figure 3) show tight coupling of results around 0 for ‘no’ classes and 1 for ‘yes’ classes, illustrating that the ensemble performs strongly on these boundaries. Inner‐range movements (considered as movement cycles within one or two meters) may share some of the characteristics of end‐range movements, such as high initial acceleration to a hitting location, offering a possible explanation for the higher rate of false positives in the current findings. The high rate of true positives in the current study indicates that end‐range movements much less commonly share attributes with inner range movements and are, therefore, less likely to be misclassified.

Despite similar F1‐scores between a single LSTM model and the ensemble, the classification of partial end‐range samples likely requires an ensemble to improve the prediction accuracy. This is apparent in the standard deviation values presented in Figure 4, where each model appears to challenge the overall prediction more so in the ‘partial’ class than ‘yes’ or ‘no’ classes. By using an ensemble, practitioners can be confident that a partial end‐range movement cycle classified as end‐range has been assessed by a variety of different models to ultimately reach this classification of end‐range. Conversely, those partial cases that are less attributable to end‐range are less likely to be classified when more models within the ensemble do not predict the end‐range class. Furthermore, the large range of values (∼0.4–∼0.85) for the partial class supports the notion of ambiguity with these cases and further increases confidence that a prediction of each partial class as end‐range shares more similar attributes with ‘textbook’ examples of end‐range movement cycles.

Using the proposed ensemble, practitioners can precisely identify LERR instances from match‐play, which overcomes the limitations present in recent investigations (Armstrong et al. 2024a; Al‐jabery, Obafemi‐Ajayi, Olbricht, and Wunsch II 2019a). Despite the methodological limitations of previous research, LERR movements account for ∼13% of all movement cycles in men's Grand Slam tennis on hard courts (Armstrong et al. 2024a). Considering the value of moving well in this scenario, and the prevalence of LERR movements during competition (Armstrong et al. 2024a; Giles et al. 2019; Armstrong et al. 2025), a highly precise identification method was warranted, and our findings provide a viable solution. Similar classification tasks of other tennis movements (i.e., CoD events) have yielded F1‐scores ranging between 0.65–0.73, with false positive rates of 17.3%–38.6% and false negative rates of 14.6%–23.7% (Giles et al. 2020). Comparatively, we observed false positive rates of < 5% (∼8‐times lower than the CoD event detection model) and false negative rates of < 9% (∼2‐times lower than the CoD event detection model) (Giles et al. 2020). Although CoD and LERR may differ, the enriched pose model data and our unique approach of using an ensemble of LSTM models may be better suited to movement related classification tasks in tennis, supported by the substantially higher F1‐score and lower rates of false positives/false negatives observed in the current investigation.

End‐range movements in tennis (akin to LERR) can compromise hitting technique and require substantial energetics (Giles and Reid 2021; Osgnach et al. 2010; Harper et al. 2022). Better ranked athletes can hit higher quality shots when peak velocity is increased during LERR movement cycles compared to their lower ranked counterparts (Armstrong et al. 2025). As such, evaluating an athlete's performance during LERR movement cycles in competition is an advantage, whereby coaches and support staff can critique an athlete's movement, tactics, and technique to enhance their performance. Until now, the industry has been bereft of an effective and precise way of identifying LERR occurrences during competition. The findings of this investigation overcome this challenge and provide a practical method for performance analysts to identify specific LERR instances, which can facilitate a timely analysis of such a critical movement cycle for professional tennis performance.

Load monitoring is a challenge in tennis competition, mostly due to competition consisting of short repeated movements whereby physiological measures (i.e., heart rate) and summarised information (i.e., high‐speed running, metres‐per‐minute) can under‐represent true physical strain (M. Kovacs 2006). Other sports have sought to enhance load quantification by including distinct events (i.e., tackles/collisions, acceleration/deceleration counts) to better inform load monitoring processes (Cummins and Orr 2015; Gastin et al. 2019; Hulin et al. 2017; Naughton et al. 2020). Ultimately, this yields improved preparation strategies, helps exploit tactics and better informs recovery requirements (Cummins and Orr 2015; Gastin et al. 2019; Hulin et al. 2017; Naughton et al. 2020). Identifying distinct events from competition in tennis proposes unique challenges. However, the findings of the current investigation make accurate identification of LERR movement in competition possible. The number of LERR efforts and athlete completes, their success when LERR efforts are required, and the quality of the shot played during LERR can all be tracked longitudinally and in a timely way with an LSTM ensemble combined with current analysis practices.

To the authors' knowledge, this is the first investigation to use pose data from competition to analyse performance in tennis. The pose model data utilised in the current investigation comes directly from athlete competition and can provide substantial kinematic movement detail from an ecologically valid setting. Pose data have historically been limited to laboratory settings, often collected with marker‐based systems, but the markerless motion system utilised in this study allows ecologically valid investigations of competitive performance, where substantial research is expected to ensue. Further to load monitoring, descriptions of specific match demands can improve athlete preparation strategies. For example, the ankles and knees attenuate most of the eccentric forces during high intensity horizontal deceleration, and insufficient preparation to handle such loads can increase an athlete's risk of injury (Harper et al. 2022). Using the pose data and the LSTM ensemble classification approach, performance staff can evaluate technical execution of deceleration biomechanics to enhance force attenuation, improving performance and lowering the risk of injury to athletes (Harper et al. 2022; M. S. Kovacs et al. 2015).

In identifying LERR instances from competition, further kinematic analysis of movement strategies and hitting techniques using pose data become more readily accessible. Such insights would help understand the footwork kinematics and loading conditions of tennis, a current gap among tennis strength and conditioning experts, who have acknowledged an inability to describe the lower limb mechanical loads and joint positions when players move in scenarios such as LERR (Lester et al. 2023). Furthermore, understanding joint angles, segment sequencing and mechanical loads during LERR motion may advance the specificity of exercise design as well as our knowledge of why better ranked players are able to express higher physical qualities and execute higher quality shots during this movement (Armstrong et al. 2025). Although the use of valid pose models is in its infancy in professional sport, using these data to describe biomechanical traits of world class tennis movers could provide movement frameworks for developing athletes to adopt. The deceleration profile of team sport athletes has included technical ability, detailing joint positions and segmental timing of high‐quality horizontal deceleration efforts which outline a deterministic model of deceleration performance (Harper et al. 2022). However, tennis movement is unique, where sliding and dissociation of upper and lower limbs are often observed. This means the deceleration profile of team sport athletes may not be applicable for tennis athletes (Carvalho et al. 2014). As such, after identifying LERR instances, further analysing pose model data can be of use in describing the most effective movement strategies in LERR deceleration, hitting and re‐acceleration.

Several limitations of this work should be considered. Firstly, our approach is dependent on three‐dimensional pose model data, the likes of which are not readily available in all competition and training venues. A more accessible approach might involve the use of match video footage or wearable inertial sensors as the input data source; however, work would be needed here to explore the efficacy of such approaches. Further, differences in movement are acknowledged between sexes, juniors, and court surfaces (Pluim et al. 2023; Ferrauti et al. 2013), which may limit the application of the current findings to females, juniors, or competition surfaces of clay or grass. Accordingly, further research is needed to understand how these factors change LERR movement and necessitates customised ensembles of LSTM models in each context. Lastly, interpretation of model classification decisions (i.e., determining which aspects of joint motions most determine end‐range movements) is difficult with LSTM models. Future work could use neural conductance and Shapely values to determine which key input features lead to such modelling classification decisions, which may be useful to further understand the partial classification more specifically (Dabounou and Baazzouz 2024; Rozemberczki et al. 2022).

## 5 Conclusion

The ability to identify the most demanding movement pattern in tennis match‐play is important to understand the physical requirements of competition and allowing the evaluation of movement characteristics that differentiate elite performers. The first step in this process is to identify each movement occurrence, which has previously been achieved through manual annotation of match footage by coach's post‐match. This is the first investigation to describe the architecture of an ensemble of LSTM models to identify instances of LERR movement cycles during match‐play, with the proposed approach achieving an F1‐score of 0.944. The findings from this investigation facilitate load monitoring practices in identifying and quantifying LERR instances during match‐play at scale, with end‐user efficiency.

## Funding

The authors received no specific funding for this work.

## Ethics Statement

The University of Western Australia research ethics committee provided ethical approval for the study (2022/ET000216).

## Consent

Consent for data collection and analysis for this study was obtained prior to each tournament at time of entry.

## Conflicts of Interest

The authors declare no conflicts of interest.
